# Emergence and dissemination of *bla*_KPC-31_ and *bla*_PAC-2_ among different species of Enterobacterales in Colombia: a new challenge for the microbiological laboratories

**DOI:** 10.1128/spectrum.01805-25

**Published:** 2025-10-10

**Authors:** Betsy E. Castro, Elsa De la Cadena, Janneth J. Escobar-Arcos, Juan C. García-Betancur, Natalia Restrepo-Arbeláez, Christian Pallares, María J. López, Angela Pescador, Juan Escobar T., Lorena Matta-Cortes, Adriana C. Palacios-Larrota, María Virginia Villegas

**Affiliations:** 1 Grupo de Investigaciones en Resistencia Antimicrobiana y Epidemiología Hospitalaria (RAEH), Universidad El Bosque 28009, Bogotá D.C., Colombia; 2 Gestión Pre-Transfusional Laboratorio y Patología, Clínica Imbanaco Grupo Quirónsalud, Cali, Colombia; 3 Comité de Infecciones y Vigilancia Epidemiológica, Clínica Imbanaco Grupo Quirónsalud, Cali, Colombia; 4 Servicio de infectología, Centro de Tratamiento e Investigación sobre el Cáncer Luis Carlos Sarmiento Angulo (CTIC) 712604 , Bogotá, Colombia; 5 Grupo de Investigaciones GIGA, CTIC/ Universidad El Bosque 28009, Bogotá, Colombia; 6 Grupo de Investigación en Medicina Cardiovascular y Especialidades de Alta Complejidad, Fundación Clínica SHAIO, Bogotá, Colombia; 7 Hospital Federico Lleras Acosta, Ibagué, Colombia; 8 Universidad del Valle 28006, Cali, Colombia; 9 Hospital Universitario del Valle 502979 , Cali, Colombia; 10 Departamento de patología y medicina del laboratorio, Fundación Valle del Lili 67597, Cali, Colombia; Universita degli Studi dell'Insubria, Varese, Italy

**Keywords:** ceftazidime/avibactam, Colombia, blaKPC-31, blaPAC-2, blaKPC-8, KPC-variants

## Abstract

Ceftazidime/avibactam (CZA) is a promising treatment option for infections caused by carbapenem-resistant Enterobacterales (CRE). However, CZA resistance is increasingly reported worldwide, largely due to the emergence of KPC variants and increase of metallo-β-lactamases (MBL). This study describes the mechanisms associated with CZA resistance in circulating Enterobacterales isolates from Colombia, highlighting the challenge this represents for microbiological identification. Between 2021 and 2024, 68 CZA-resistant Enterobacterales isolates were identified by automated methods in seven Colombian cities. Resistance to CZA was subsequently confirmed by broth microdilution and E-test. Carbapenemase production was evaluated using phenotypic tests, such as the mCIM test, Carba NP, lateral flow assay, and qPCR (*bla*_KPC_, *bla*_NDM_, *bla*_VIM_, *bla*_IMP_, and *bla*_OXA-48_). Whole-genome sequencing was performed on 15 isolates that tested negative for MBL genes. Whole-genome sequencing of these 15 isolates revealed a variety of resistance determinants: six isolates harbored *bla*_KPC-31_, one *bla*_KPC-33_*,* one *bla*_KPC-8_, five harbored *bla*_PAC-2_, and two co-harbored *bla*_PAC-2_ and *bla*_KPC-2_. Notably, *bla*_PAC-2_ was located on an IncQ plasmid. However, some of these variants were not detected by phenotypic assays, likely due to their low or undetectable carbapenemase activity. CZA resistance in non-MBL producing Enterobacterales in Colombia is primarily mediated by the presence of *bla*_KPC-31_ and emergence of *bla*_PAC-2_. These resistance mechanisms pose significant diagnostic, therapeutic, and epidemiological challenges, as they frequently go undetected by conventional microbiological methods. In this context, enhanced molecular surveillance and improved diagnostic strategies are urgently needed to enable early detection, guide antimicrobial therapy, and support infection control and stewardship efforts.

**IMPORTANCE:**

Antibiotic resistance is a serious global health threat. Ceftazidime/avibactam (CZA) is a key treatment option for multidrug-resistant (MDR) Enterobacterales often used when other antibiotics fail. However, bacteria are now developing resistance to this drug as well, making infections increasingly difficult to treat. In this study, we examined CZA-resistant bacteria from multiple cities in Colombia and found uncommon resistance genes across several bacterial species. These genes are frequently missed, as they often do not test positive due to the limitations of most routinely used laboratory tests. Importantly, some of these genes can be transferred between bacteria, increasing the likelihood of indiscriminate dissemination in the hospital setting. Therefore, our findings highlight the urgent need for improved diagnostic tools and molecular surveillance. Early detection will help physicians select effective treatments quickly and prevent the wider dissemination of these MDR-resistant bacteria.

## INTRODUCTION

Resistance to carbapenems can be mediated by several mechanisms; in carbapenem-resistant Enterobacterales (CRE), the most common is the production of carbapenemases (1). The most frequent carbapenemases worldwide in CRE are KPC, NDM, and OXA-48-like, which can spread rapidly within different bacterial species facilitating their dissemination (2, 3). One of the few therapeutic options available for the treatment of CRE infections in Latin America (LATAM) countries is ceftazidime/avibactam (CZA) (4). Avibactam is an innovative β-lactamase inhibitor of class A, C, and some class D β-lactamases (Ambler classification). However, CZA is not active against class B β-lactamases, such as NDM, VIM, or IMP (5). Some *in vitro* studies have demonstrated that avibactam can restore the antimicrobial activity of ceftazidime against many isolates that harbor extended-spectrum β-lactamase (ESBL) producing Enterobacterales, AmpC β-lactamases, *Klebsiella pneumoniae* carbapenemases (KPC), and OXA-48-like carbapenemases. In the case of carbapenemases, avibactam acts as a reversible inhibitor that forms a covalent bond with the active-site serine of the β-lactamase, which slowly dissociates, allowing the inhibitor to regenerate its original structure preventing the hydrolysis (6).

Globally, CZA resistance rates in non-metallo-β-lactamases (MBL) producing Enterobacterales remain low (7). Unfortunately, the emergence of novel mutations in genes, including CMY, CTX-M-15/14, GES-19/26, and KPC-2/3, is increasingly relevant to the development of resistance to CZA (8–10). Mutations in the active site (Ω-loop) of carbapenemases, such as KPC, are also important, as they disrupt a critical salt bridge between Arg164 and Asp179, which is essential for maintaining the integrity of the loop’s cyclic structure. Mutations occurring in the site generate flexibility of the Ω-loop, which enhances covalent trapping of β-lactamases to CAZ and decreases avibactam binding, causing resistance to CZA (8). In some cases, this diversity of structural changes leading to CZA resistance (mainly driven by Ω-loop mutations) preserves the carbapenemase profile, while in others, it restores *in vitro* susceptibility to carbapenems while maintaining a cephalosporinase profile (11, 12). In this context, the interpretation of susceptibility to carbapenems in the presence of carbapenemase mutations, especially KPC variants, poses a challenge for microbiology laboratories, especially in highly endemic areas of AMR, such as LATAM, where multiple resistance mechanisms coexist. This is the case for the KPC-31 variant, which does not hydrolyze carbapenems and is not detected by screening tests like the mCIM test, Carba NP, and lateral flow immunoassays (9). Additionally, the limited resources for molecular diagnosis do not allow adequate monitoring of CZA resistance and the resistance mechanisms involved (13).

In addition to the presence, diversity, and dissemination of carbapenemases, a novel mechanism associated with resistance to CZA was identified back in 2010 in India, with the report of the *bla*_PAC-1_ gene coding for a β-lactamase of class C. Up to recent years, its dissemination has been limited to sporadic cases, primarily in *Pseudomonas aeruginosa* ST664 isolates of both clinical and animal origin from Nepal and Singapore, as well as in environmental isolates of *Aeromonas caviae* recovered from water reservoirs. In these isolates, the presence of *bla*_PAC-1_ has been located exclusively on the chromosome, within a complex Tn1721-like transposon (14, 15). Recently, the National Institute of Health of Colombia (INS) reported the recovery of a CZA-resistant *Klebsiella quasipneumoniae* carrying a *bla*_PAC-1_ variant, designated *bla*_PAC-2_ (GenBank ID: PQ567105.1) which to date represents*,* to the best of our knowledge, the only sequence available (12).

Moreover, the emergence of novel resistance mechanisms presents a significant challenge for laboratory diagnosis. The PAC enzyme hydrolyzes penicillins and first and second generation cephalosporins, but not third and fourth generation cephalosporins or carbapenems (14, 15); however, avibactam seems to have no inhibitory activity against PAC-1, which hydrolyzes ceftazidime even in the presence of the inhibitor. Consequently, PAC activity and its phenotype cannot be accurately detected by standard susceptibility methods, and due to the limited availability of advanced molecular tools, such as targeted PCR or whole-genome sequencing (WGS), in most clinical laboratories, these isolates may be completely overlooked (16). From a laboratory perspective, accurately interpreting CZA resistance and effectively reporting these results to physicians presents a significant challenge, which is critical for guiding timely and appropriate therapeutic decisions along with conducting epidemiological surveillance. This study describes the molecular mechanisms found in non-MBL CZA-resistant Enterobacterales isolates in Colombia and the challenges associated with their accurate identification.

## MATERIALS AND METHODS

### Isolate collection and phenotypic testing

Between September 2021 and September 2024, as part of a multicenter nationwide surveillance study to track CZA resistance, 68 unduplicated CZA-resistant clinical isolates of Enterobacterales identified by automated methods were recovered from adult patients in 20 healthcare institutions across seven Colombian cities (Medellín, Cali, Bogotá, Cartagena, Ibagué, Manizales, and Rionegro). These isolates (*K. pneumoniae*, *n* = 46; *Escherichia coli*, *n* = 10; *Enterobacter cloacae*, *n* = 2; *Serratia marcescens*, *n* = 3; *Proteus mirabilis*, *n* = 2; *Klebsiella oxytoca*, *n* = 1; *Kluyvera ascorbata, n* = 1; *Salmonella* sp., *n* = 1; *Enterobacter hormaechei, n* = 1, and *Citrobacter freundii*, *n* = 1) were sent to our laboratory for confirmation. Resistance to CZA and other antibiotics was initially performed by broth microdilution (BMD) test using Sensititre, customized panels (Thermo Fisher Diagnostics, USA) and ETEST gradient diffusion tests (Marcy-l’Etoile, France) following the manufacturer’s instructions. The results were interpreted according to the Clinical and Laboratory Standards Institute (17) and the U.S. Food and Drug Administration guidelines for tigecycline (18). *K. pneumoniae* ATCC 700603*, K. pneumoniae* carbapenemase (KPC) producer ATCC BAA-1705, and *E. coli* ATCC 25922 were used as quality control strains. The isolates with confirmed CZA resistance were evaluated for carbapenemase production by phenotypic tests, such as mCIM, lateral flow immunoassays (LFIA) NG Test CARBA5 (NG-Biotech, Guipry, France) and CARBA-NP (17, 19). Additionally, the presence of the *bla*_KPC_, *bla*_NDM_, *bla*_VIM_, *bla*_IMP_, and *bla*_OXA-48-like_ genes was evaluated using multiplex qPCR (20).

### Genomic analysis

Isolates with confirmed CZA resistance and no detectable MBLs were selected for WGS, using NextSeq 1000 technology (Illumina Inc., USA). Genomic DNA was extracted with the UltraClean kit (Qiagen, Germany) of overnight cultures. The raw genomic data obtained was assembled *de novo* using SPADES with predetermined parameters (21). Open reading frames (ORF) annotation was done in Bakta v1.13.28 (22) and on the RAST server (23). Bacterial characterization included species identification (SpeciesFinder), multilocus sequence typing (24), antibiotic resistance gene determination (ResFinder and CARD) (25, 26), capsular typing (wzi gene), and virulence gene typing using Kleborate for *K. pneumoniae* (27). The incompatibility group (Inc.) was defined based on the genes of the replicon (PlasmidFinder) (28). The main outer membrane porins OmpK35 and OmpK36 were identified *in silico* and compared with reference wild-type sequence from *K. pneumoniae* ATCC 43816 using BLASTp. To establish the transfer capacity of the *bla*_PAC-2_ gene, a conjugation assay was performed using a 1:4 ratio in Bertani Luria broth (LB) of the donor isolates *K. pneumoniae* (1381), *K. ascorbata* (1385), and *E. hormaechei* (1387) and the strain of *E. coli* J53^AziR^ strain as a recipient. Transconjugants (TC) were selected in LB supplemented with 100 µg/mL of sodium azide and 16 µg/mL of ceftazidime (29). To assess whether the STs of our isolates were related to previously reported CZA-resistant strains, we analyzed assembled genomes from the NCBI Pathogen Detection Repository, using species and the presence of the KPC-8, KPC-31, and KPC-33 variants as search criteria (accessed August 2025). The STs of each genome were then determined (24).

## RESULTS

**Fig 1:**
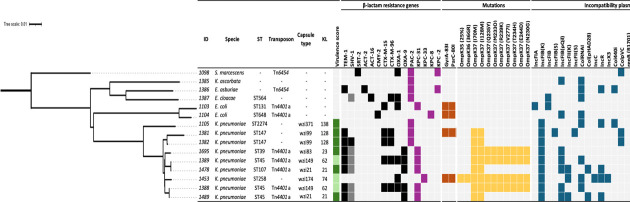
Mash distance tree of CZA-resistant isolates. Virulence score for *K. pneumoniae* was performed according to the Kleborate criteria: 0 = absence of the yersinibactin (*ybt*), colibactin (*clb*), and aerobactin (*iuc*) genes indicated in green and 1 = presence of only *ybt,* indicated in light green. β-Lactam resistance genes are represented in black squares, and non-SHV-1 variants in dark gray. Genes related to CZA resistance are shown in violet, Gyr and ParC substitutions related to quinolone resistance in orange, mutations in porins OmpK35 and 37 in yellow, and incompatibility groups in blue. The absence of genes of interest or those not applicable to the species is indicated in gray. (–): data not applicable to the species.

Resistance to CZA was confirmed by ETEST and BMD in 79.4% (54/68) of the isolates, while the remaining 20.6% (14/68) were susceptible, showing discrepancies with the automated methods performed in the healthcare institutions. From the 54 confirmed CZA-resistant isolates, 28/54 isolates (51.9%) were shown to co-harbor the *bla*_NDM_ and *bla*_KPC_ genes, 1/54 (1.8%) co-harbored the *bla_KPC_* and *bla*_VIM_ genes, and 10/54 (18.5%) carried *bla*_NDM_. On the other hand, 8/54 (14.8%) isolates harbored only *bla*_KPC_, and 7/54 (13.0%) did not carry any of the carbapenemase genes evaluated. These 15 isolates represented six species of which *K. pneumoniae* was the most frequent (9/15, 60%). All 15 isolates underwent sequencing to identify the mechanism of resistance. Based on the WGS results, 6/15 (40.0%) isolates were found to be positive for *bla*_KPC-31_, 1/15 (6.7%) for *bla*_KPC-33_, and 1/15 (6.7%) for *bla*_KPC-8_. These isolates were obtained from patients from different cities (Bogotá, Cali, and Ibagué). In the remaining isolates, 5/15 (33%) harbored a plasmidic *bla*_PAC-2_ gene, and 2/15 (13.4%) isolates co-harbored the *bla*_PAC-2_ and *bla*_KPC-2_ genes (Fig. 1 and Table 1). It should be noted that all the *bla*_PAC-2_ isolates came from the same healthcare institution in Cali. WGS analysis identified six different sequence types (ST) for the *K. pneumoniae* clinical isolates, including ST45 (*n* = 2), ST147 (*n* = 2), ST107 (*n* = 2), ST39 (*n* = 1), ST2274 (*n* = 1), and ST258 (*n* = 1); two different ST for *E. coli* ST131 (*n* = 1) and ST648 (*n* = 1); and one ST from *E. cloacae* ST564 (*n* = 1) which partially correspond to the lineages of the previously reported isolates (Fig. 1; Table S1).

**TABLE 1:** Phenotypic characterization of CZA-resistant Enterobacterales

ID	Specie^*a*^	City	Isolation source	Collection date	CZA (E-test)	C/T	CZA	AZT	CAZ	FEP	PTZ	ERT	IMI	MER	AMK	CIP	TGC	ESBL	LFIA	CARBA NP	mCIM	GEN
1105	Kpn	Cali-H1	ND	2023	128	8 R	128 R	≤1 S	≥64 R	≤0.12 S	8 S	≤0.12 S	≤0.25 S	≤0.25 S	≤1 S	≤0.006 S	≤0.5 S	−	NA	NA	NA	PAC-2
1381	Kpn	Cali-H1	Urine	2024	>256	≥32 R	≥16 R	32 R	≥64 R	≥32 R	32 R	≤0.12 S	≤0.25 S	≤0.25 S	≤1 S	8 R	≥8 R	−	NA	NA	NA	PAC-2
1382	Kpn	Cali-H1	Blood	2024	>256	8 R	≥16 R	≤1 S	≥64 R	1 S	≤4 S	≤0.12 S	≤0.25 S	≤0.25 S	≤1 S	≤0.006 S	≤0.5 S	+	NA	NA	NA	PAC-2
1385	Kas	Cali-H1	Blood	2024	>256	≥32 R	≥16 R	16 R	≥64 R	2 S	16 I	≤0.12 S	0.5 S	≤0.25 S	2 S	0.5 S	≤0.5 S	NA	NA	NA	NA	PAC-2
1387	Eho	Cali-H1	Peritoneal f.	2024	>256	≥32 R	≥16 R	≥64 R	≥64 R	≥32 R	64 R	≤0.12 S	0.5 S	≤0.25 S	4 S	0.5 S	≤0.5 S	NA	NA	NA	NA	PAC-2
1098	Sma	Cali-H1	Urine	2023	>256	≥32 R	≥16 R	32 R	≥64 R	>16 R	32 R	≤0.12 S	≤0.25 S	≤0.25 S	≤1 S	≥4 R	≥8 R	NA	+	+	+	KPC-2/PAC-2
1386	Eas	Cali-H1	ND	2024	>256	≥32 R	≥16 R	16 R	≥64 R	≥32 R	≥128 R	≥8 R	4 R	1 S	2 S	2 R	≤0.5 S	NA	+	+	+	KPC-2/PAC-2
1103	Eco	Bogota-H2	ND	2023	>256	≥32 R	≥16 R	16 R	≥64 R	≥32 R	≥128 R	≥8 R	8 R	≥16 R	4 S	≥4 R	≤0.5 S	+	−	−	−	KPC-31
1388	Kpn	Cali-H1	Blood	2024	>256	≥32 R	≥16 R	≥64 R	≥64 R	≥32 R	32 R	0.5 S	≤0.25 S	≤0.25 S	2 S	≥4 R	≤0.5 S	+	−	−	−	KPC-31
1389	Kpn	Cali-H1	Blood	2024	>256	≥32 R	≥16 R	≥64 R	≥64 R	≥32 R	64 R	0.25 S	≤0.25 S	≤0.25 S	32 R	≥4 R	≤0.5 S	+	−	−	−	KPC-31
1478	Kpn	Bogota-H3	Secretion	2024	>256	≥32 R	≥16 R	≤1 S	≥64 R	2 S	≤4 S	≤0.12 S	≤0.25 S	≤0.25 S	≤1 S	≤0.006 S	≤0.5 S	−	−	−	−	KPC-31
1489	Kpn	Ibague H4	Secretion	2024	>256	≥32 R	≥16 R	≥64 R	≥64 R	≥32 R	≥128 R	≥8 R	≥16 R	≥16 R	16 R	≥4 R	1 S	−	−	−	−	KPC-31
1695	Kpn	Cali-H5	Blood	2024	>256	≥32 R	≥16 R	2 S	≥64 R	≥32 R	≥128 R	≥8 R	0.5 S	4 R	4 S	≥4 R	2 S	−	−	−	−	KPC-31
1453	Kpn	Cali-H6	ND	2024	>256	≥32 R	≥16 R	≥64 R	≥64 R	≥32 R	≥128 R	≥8 R	8 R	≥16 R	32 R	≥4 R	1 S	−	−	+	+	KPC-33
1104	Eco	Cali-H5	ND	2023	32	≥32 R	≥16 R	≥64 R	≥64 R	≥32 R	≥128 R	≥8 R	≥16 R	≥16 R	4 S	≥4 R	≤0.5 S	−	+*	+	+	KPC-8

^*a*^ Kpn*, K. pneumoniae*; Eco, *E. coli*; Eho, *E. hormaechei*; Sma, *S. marcescens*; Eas, *Enterobacter asburiae*; Kas, *K. ascorbata*; H, hospital; C/T, ceftolozane/tazobactam; CZA, ceftazidime/avibactam; IMR, imipenem relebactam; MEV, meropenem vaborbactam; AZT, aztreonam; CAZ, ceftazidime; FEP, cefepime; PTZ, piperacillin/tazobactam; ERT, ertapenem; IMP, imipenem; MER, meropenem; AMK, amikacin; CIP, ciprofloxacin; TGC, tigecycline; S, susceptible; I, intermediate; R, resistant; ESBL, extended-spectrum B-lactamase; LFIA, lateral flow immunoassays; mCIM, modified carbapenemics inactivation method. (+) positive; (−) negative; NA, not applicable. (*): in the LFIA assay a weak band was observed. The values in the boxes indicate the MIC value in µg/mL. The MIC90 of CZA were 256 µg/mL.

In *K. pneumoniae*, the genomic environment for the carbapenemase gene *bla*_KPC-8_ and *bla*_KPC-31_ was clearly associated with a typical Tn*4401a* structure, composed of two insertion sequences, ISKpn7 and ISKpn6, a transposase gene (*tnpA*) and a resolvase gene (*tnp*R), while the *bla*_KPC-2_ gene was present in a Tn*6454* structure. The 1103-*E. coli bla*_KPC-31_ was associated with the high-risk clone ST131-*H30*-Rx, which also harbored *bla*_CTX-M-15_ and mutations in QRDR (region determining quinolone resistance), *GyrA*-83I and *ParC*-80I confer resistance to ciprofloxacin.

**Fig 2:**
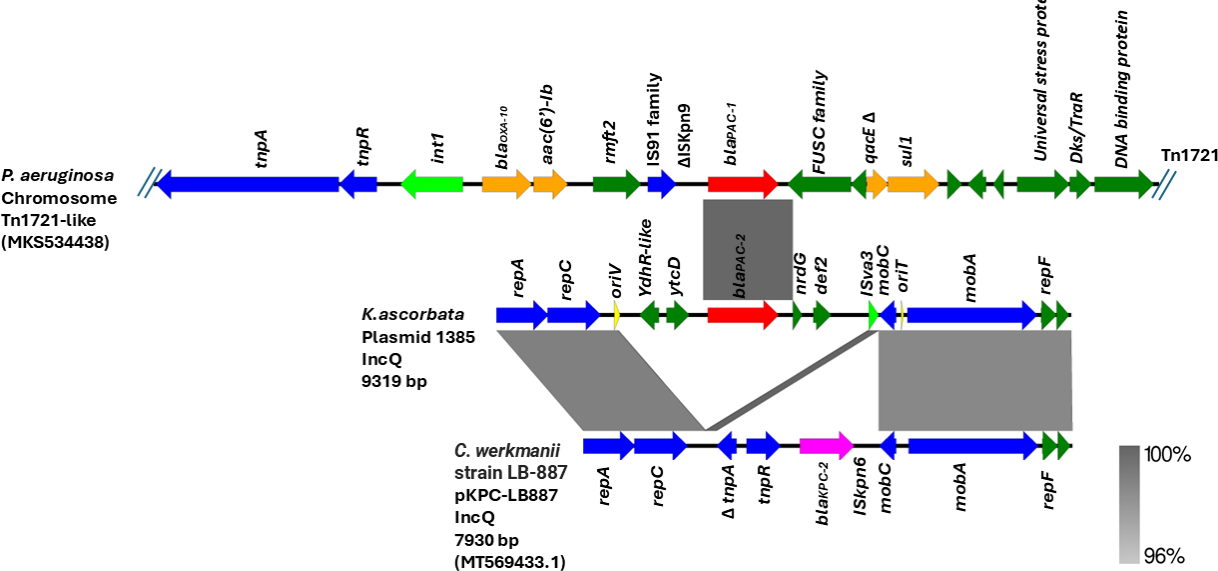
Genetic environment of *bla_PAC_* genes. Comparison of the chromosomal genomic environment of *bla*_PAC-1_ from *P. aeruginosa* to the IncQ plasmid harboring *bla*_PAC-2_ present in *K. ascorbata* and pKPC-LB887 plasmids harboring KPC-2 in a *Citrobacter werkmanii* isolate from coastal water that share the plasmid backbone. The shaded area between the sequences delimits the alignment regions with a percentage of identity ≥96%. The arrows indicate the open reading frames: the *bla*_PAC_ gene is shown in red, other resistance genes in orange, and genes related to plasmid mobilization and conjugation in blue.

Of major interest, the *bla*_PAC-2_ gene, conferring resistance to CZA, was identified in five different species of Enterobacterales: *K. pneumoniae* (*n* = 3), *E. hormaechei* (*n* = 1), *K. ascorbata* (*n* = 1), *S. marcescens* (*n* = 1), and *Enterobacter asburiae* (*n* = 1). Interestingly, these last two species co-harbor *bla*_KPC-2_ and *bla*_PAC-2_ genes. The genomic analysis of this *bla_PAC-2_* gene revealed three amino acid substitutions when compared to the bla_PAC-1_ gene (K44Q, E309A, and N339K) and was first detected on an IncQ plasmid with a size of 9.3 Kb (Fig. 2), conserved in all bacterial species analyzed in this study. This plasmid IncQ was conjugated in *E. coli* J53^azR^ from the donor strains *K. pneumoniae* (1381), *K. ascorbata* (1385), and *E. hormaechei* (1387), which suggests ability to dissemination in different hosts (Table 2).

**TABLE 2:** Resistance profile of parental and TC strains

Antibiotics	J53_*E. coli*	Kpn_1381	TC_1381	Kas_1385	TC_1385	Eho_1387	TC_1387
CAZ	≤0.12	≥64	≥64	≥64	≥64	≥64	≥64
CZA	≤0.12	≥16	≥16	≥16	16	≥16	≥16
C/T	≤0.25	≥32	2	≥32	2	≥32	4
AZT	≤1	32	16	16	4	≥64	32
FEP	≤0.12	≥32	2	2	2	≥32	8
PTZ	≤0.12	32	32	16	16	64	32
ERT	≤0.12	≤0.12	≤0.12	≤0.12	≤0.12	≤0.12	≤0.12
IMI	≤0.25	≤0.25	≤0.25	0.5	≤0.25	0.5	≤0.25
MER	≤0.25	≤0.25	≤0.25	≤0.25	≤0.25	≤0.25	≤0.25
AMK	2	≤1	2	2	2	4	2
CIP	≤0.06	≥8	≤0.06	0.5	0.5	0,5	≤0.06
TGC	≤0.5	≥8	≤0.5	≤0.5	≤0.5	≤0.5	≤0.5

Detailed analysis of the IncQ circularized plasmid harboring the *bla*_PAC-2_ gene did not show any additional resistance or virulence genes; instead, it showed the presence of the *repA* gene related to plasmid replication and the *mob* genes that allow transfer during conjugation to a broad-host-range. When comparing the genetic environment of the *bla*_PAC-2_ gene, no evident similarity was found with the genomic environment of the *bla*_PAC-1_ identified in isolates of *P. aeruginosa* (GenBank ID: MK534438) and *A. caviae* (GenBank ID: PUTR01000038.1) (Fig. 2). Furthermore, the chromosomal environment of the *bla*_PAC-1_ gene in *P. aeruginosa* is comprised of a cluster of genes harboring a class 1 integrase Δ*SKpn9 -bla*_PAC-1_*-2orfs -qacEΔ1-sul1* present in Tn*1721*-like, suggesting that the acquisition of *bla*_PAC-2_ on the IncQ plasmid is not mediated by this transposon (Fig. 2). However, IncQ plasmids identified in the isolates of the study show high similarity with other IncQ plasmids found in *Citrobacter werkmanii* (Sequence ID: MT569433.1) and *Aeromonas* sp. (GenBank ID: CP187599.1) containing *bla*_KPC-2_ in the same plasmid with a similarity coverage of 58% and identity of 96.6%, which do not harbor additional resistance genes (Fig. 2).

The 15 isolates sequenced with *bla*_KPC-31,_ *bla*_KPC-33_, *bla*_KPC-8_, and *bla*_PAC-2_ were subjected to the BMD test. The MIC of carbapenems from isolates with *bla*_KPC-31_ was variable. Isolates producing only *bla*_PAC-2_ were susceptible to all carbapenems but showed variable susceptibility to cephalosporins and piperacillin–tazobactam. Both isolates (1098 and 1386) harboring KPC variants and the isolates with PAC-2 were fully susceptible to imipenem relebactam and meropenem vaborbactam (Table 1). Among the *bla*_KPC-31_ isolates, both Carba-NP and LFIA assays showed negative results. In contrast, *bla*_KPC-33_ was positive for mCIM and Carba-NP, and the LFIA assay was negative, while *bla*_KPC-8_ was positive, but the test band was perceptively weaker than the control band (Table 1). In addition, WGS allowed us to enable the identification of nonsynonymous mutations previously reported occurring in carbapenem-resistant strains in OmpK36 (36GR) and OmpK37 (Q235Y, M233Q, R239K, V277I, T234H, E244D, N230G, I70M, I128M) (Fig. 1). Additionally, nonsynonymous mutations in DNA gyrase GyrA (S83I) and topoisomerase IV ParC (S80I) were detected, primarily in *K. pneumoniae* isolates and are responsible for acquired resistance to fluoroquinolones (30).

## DISCUSSION

The emergence and dissemination of CZA-resistant clinical isolates represents a major therapeutic concern, particularly in countries with high prevalence of CRE (31). Since the introduction of CZA in 2015, numerous studies have reported resistance mediated by KPC variants, particularly mutations within the Ω-loop region which reduce susceptibility to avibactam while maintaining hydrolytic activity against ceftazidime (8). In our study, the results highlighted that CZA resistance in Enterobacterales from various cities and species in Colombia is primarily mediated by KPC variants (*bla*_KPC-31_, *bla*_KPC-33_, and *bla*_KPC-8_), as well as the cephalosporinase gene *bla*_PAC-2_.

In Colombia, CZA was approved for clinical use in 2020, specifically for the treatment of complicated urinary tract infection, intra-abdominal, nosocomial pneumonia, and sepsis caused by CRE. To date, more than 200 *bla_KPC_* variants have been reported worldwide (https://www.ncbi.nlm.nih.gov). Most of these variants originate from the *bla*_KPC-2_ and *bla*_KPC-3_ genes, which are the most prevalent carbapenemases globally. For instance, *bla_KPC-_*_31_ and *bla_KPC-8_* are derived from *bla*_KPC-3_, while *bla*_KPC-33_ originates from *bla*_KPC-2_. However, it is important to note that not all KPC variants confer resistance to CZA (32). The distinct enzymatic profiles of these variants result in differential susceptibility patterns to β-lactam–β-lactamase inhibitor combinations, with several exhibiting cross-resistance both to CZA and carbapenems (33). In 2024, two CZA-resistant *K. pneumoniae* belonging to ST45 and ST258 were reported for the first time in Colombia: one harboring the *bla*_KPC-197_ gene with the A177E substitution, a deletion (168-169_EL) within the Ω loop and the insertion of two amino acids at position 274 (Ins_274_DS), the other isolate carried a *bla*_KPC-31_ with mutations in the Ω loop (D179Y) just like *bla*_KPC-33_ (34). These mutations are associated with increased ceftazidime hydrolysis and reduced avibactam inhibition (9, 11).

In this study, the CZA-resistant *E. coli* isolate 1103, carrying *bla*_KPC-31_, belonged to ST131, which is worrisome because of the high probability of dissemination in the community (35). This lineage is recognized worldwide as an important clone associated with the spread of the ESBL CTX-M-15 in extraintestinal *E. coli,* being ST131 *bla*_CTX-M-15_ a key driver of third-generation cephalosporin resistance (36). In Colombia, about 28% of *E. coli* isolated from in-hospital settings are resistant to third-generation cephalosporins, according to recent data (37). In contrast, carbapenem resistance in *E. coli* remains particularly low, with reports indicating a resistance rate of 1%–2%. Our study presents, this is, to our knowledge, the first report of *bla*_KPC-8_ in Colombia associated with *E. coli* (1104 isolates). This variant was previously identified in Puerto Rico in 2008 and has been mainly associated with *K. pneumoniae* belonging to ST512 and ST258 (Table S1) (38). This variant emerged before the introduction of CZA and is not inhibited by avibactam, reflecting a distinct evolutionary path compared to more recently emerged variants and raising concerns about international dissemination (35, 39).

On the other hand, different KPC variants have distinctive antimicrobial susceptibility phenotypes. The majority exhibit resistance to CZA and enhanced hydrolytic activity toward ceftazidime, like the case of *bla*_KPC-31_ (9), while other variants reduce the inhibition by avibactam (32, 33). Most KPC variants will show a reversed susceptibility to carbapenems (especially imipenem and meropenem), limiting the accuracy of phenotypic methods for carbapenemase detection, such as Carba-NP assay, mCIM, and LFIA (13, 19). The false-negative results from these commonly used tests to detect carbapenemases will result in the limitation of microbiology laboratories to identify resistant isolates, delaying prescription of effective treatment to identify resistant isolates, delaying prescription of effective treatment options. Moreover, certain KPC mutations that reduce carbapenem affinity and thus result in susceptibility to imipenem and meropenem have been reported to be variable. During treatment, these mutations can revert, leading to loss of carbapenem resistance (13). As previously described, certain variants and mutants of KPC, such as KPC-31, yield false-negative results in screening tests like LFIA and Carba-NP (9). These challenges highlight the complexity of detecting emerging resistance mechanisms, particularly in low- and middle-income countries like Colombia, where limited access to advanced laboratory tests, including genomic sequencing, hampers timely and accurate diagnosis.

Finally, this report of *bla*_PAC-2_ gene conferring resistance to CZA in clinical isolates of Enterobacterales highlights the potential for the rapid dissemination of this novel CZA resistance mechanism via a highly transmissible mobile genetic element: the *bla*_PAC-2_/IncQ. As our results indicate, this resistance was detected in five different species of Enterobacterales, suggesting an active dissemination, as well as a very successful interspecies mobilization ability. In the case of PAC-2, the cephalosporinase exhibits variable hydrolytic activity against cephalosporins and is capable of hydrolyzing last-resort antibiotics, such as CZA and ceftalozone/tazobactam. Since phenotypic testing indicates carbapenem susceptibility, the discrepancy in microbiological identification could lead to inappropriate treatment choices, potentially resulting in clinical failure. As shown in this study, phenotypic resistance to CZA was observed in both KPC mutants and PAC-2 producers; however, phenotypic profiles differ. In the case of KPC mutants, carbapenems exhibited a variable reversed susceptibility, whereas PAC-2, being a cephalosporinase, confers resistance to CZA while preserving carbapenem susceptibility. This distinction is critical for tailoring appropriate treatment regimens.

Given the limitations of current microbiological tools, phenotypic testing alone makes it difficult to detect and contain the spread of both KPC mutants and PAC-2, hindering effective infection prevention and control. Emphasis should be placed on integrating advanced molecular diagnostics into routine microbiology workflows, particularly when phenotypic discrepancies arise or in regions endemic for AMR. Addressing CZA resistance has become a pressing challenge to preserve this vital therapeutic option, especially for infections caused by Enterobacterales.

### Conclusions

In this study, we report the circulation in Colombia of KPC variants and the PAC-2 cephalosporinase, both contributing to CZA resistance across diverse, clinically relevant species of Enterobacterales. The emergence and dissemination of these resistance mechanisms in Colombia, largely considered a hotspot for AMR, pose significant challenges for accurate microbiological detection and informed therapeutic decision-making. Enhancing access to advanced diagnostic tools and prioritizing the containment of these mechanisms should be key objectives for antimicrobial stewardship programs, as well as for infection prevention and control efforts.

## Data Availability

The genomes of this study have been deposited in the GenBank under the Bioproject PRJNA1273169.

## SUPPLEMENTAL MATERIAL

Table S1 spectrum.01805-25-s0001.xlsx Genomic characteristics of KPC variant-carrying isolates.

ASM does not own the copyrights to Supplemental Material that may be linked to, or accessed through, an article. The authors have granted ASM a non-exclusive, world-wide license to publish the Supplemental Material files. Please contact the corresponding author directly for reuse.
